# Knowledge and attitudes toward cesarean scar pregnancy among post-cesarean section women

**DOI:** 10.3389/fpubh.2026.1758292

**Published:** 2026-06-15

**Authors:** Qionglan Li, Shuxia Chen, Hong Yan, Mingyuan Huang, Yuanyuan Guo, Nanxiu Gao

**Affiliations:** 1 Department of Obstetrics 5, Fujian Maternal and Child Health Hospital, Fuzhou, China; 2 Department of Obstetrics 2, Fujian Maternal and Child Health Hospital, Fuzhou, China

**Keywords:** attitude, cesarean scar pregnancy, cross-sectional study, knowledge, post-cesarean section women

## Abstract

**Objectives:**

The present study aimed to examine the knowledge and attitudes of post-cesarean section women toward cesarean scar pregnancy (CSP).

**Methods:**

The present cross-sectional study examined women who had undergone cesarean section in two Fujian hospitals from November 20, 2023, to December 30, 2023. Sociodemographic data and knowledge and attitude scores were obtained utilizing a self-designed questionnaire.

**Results:**

The 325 patients enrolled were aged 31.87 ± 4.21 years. Their knowledge and attitude scores were 11.14 ± 4.34 (range: 0 to 20) and 49.88 ± 5.79 (range: 12 to 60), respectively. In multivariable logistic regression analysis, junior college/bachelor’s degree (OR = 4.742, 95%CI: 1.399–16.069; *p* = 0.012) and Master’s degree and beyond (OR = 9.981, 95%CI: 2.251–44.255; *p* = 0.002) had independent associations with good knowledge. Additionally, high school/vocational high school education (OR = 3.534, 95%CI: 1.224–10.198; *p* = 0.020) and junior college/bachelor’s degree (OR = 12.352, 95%CI: 4.373–34.887; *p* < 0.001) had independent associations with a positive attitude.

**Conclusion:**

Post-cesarean section women may show inadequate knowledge and positive attitude toward CSP. Health professionals should provide tailored educational activities to improve awareness, particularly for women with low education.

## Introduction

Over recent decades, the global increase in cesarean section rate has precipitated a concurrent rise in associated complications. These range from immediate postoperative issues, e.g., postpartum hemorrhage and uterine rupture, to long-term repercussions such as invasive placenta, infertility, and notably, cesarean scar pregnancy (CSP) (1). CSP, a distinct and rare complication, manifests when a gestational sac implants within the scar tissue of a previous cesarean section, embedding itself either partially or fully into the uterine muscle layer (2). The 2020 European Society of Human Reproduction and Embryology guidelines categorize CSP as a unique form of ectopic pregnancy (3), with current statistics indicating a prevalence of 1.15% among women with a history of cesarean section.

CSP manifests in many ways, and some women exhibit nonspecific signs resembling a threatened abortion, e.g., minimal vaginal bleeding and mild lower abdominal pain. About 20–25% of cases show no overt symptoms at diagnosis (4, 5). However, serious complications may occur, e.g., fetal death, preterm birth, uterine rupture, substantial bleeding, and maternal death, indicating an important threat to women’s reproductive health and survival (6). Therefore, early abortion is commonly proposed for reducing the inherent risks and preventing further harm to these mothers.

The Knowledge, Attitude, and Practice (KAP) model is broadly utilized to examine and modify health-related behaviors (7, 8). Using KAP surveys to examine women who had undergone cesarean section regarding CSP may help detect symptoms early and seek timely medical attention, thereby decreasing CSP-associated complications. Additionally, patients with a positive attitude toward preventive and therapeutic approaches proactively seek medical care and show higher compliance with medical advice.

Despite the impressive understanding of CSP’s clinical importance, post-cesarean section women are scarcely assessed for their knowledge and attitudes toward CSP. Thus, the present work aimed to examine the knowledge and attitudes of post-cesarean section women toward CSP.

## Materials and methods

### Study design and participants

This cross-sectional study was conducted on women who had undergone cesarean section at the Fujian Maternal and Child Health Hospital and Fujian Provincial Maternity and Child Health Care Hospital between November 20, 2023, and December 30, 2023. Inclusion criteria for participants were as follows: (1) cesarean section within the past 7 days; (2) age of 18 to 45 years; (3) clear consciousness and capability of independently responding to the questionnaire. Women with serious underlying diseases during pregnancy—such as uncontrolled cardiovascular conditions, hepatic or renal insufficiency, active infectious diseases, or severe psychiatric disorders—were excluded to ensure maternal-fetal safety and population homogeneity. Ethical approval for the study was obtained from the Ethics Committee of our Hospital, and informed consent was obtained from all participants.

### Questionnaire introduction

The questionnaire design was informed by established guidelines of the *Society for Maternal-Fetal Medicine (SMFM) Consult Series #49: Cesarean scar pregnancy (2022)* (9), *Expert opinion of diagnosis and treatment of cesarean scar pregnancy (2016)* (10), and *Expert consensus on the midterm pregnancy termination complicated with placenta previa status and increta after cesarean section* (11). Following the initial design phase, feedback from five experts in obstetrics and gynecology (each with over 10 years of experience) was solicited and integrated, with adjustments made to enhance clarity in specific questions. A pilot study involving a small sample size (36 women) was conducted, yielding an overall Cronbach’s *α* coefficient of 0.820, which indicated good internal consistency. To further assess the structural validity of the questionnaire, a confirmatory factor analysis (CFA) was conducted. The model demonstrated a good fit (CMIN/DF = 1.630, RMSEA = 0.044, IFI = 0.917, TLI = 0.901, CFI = 0.915), supporting the construct validity of the knowledge and attitude dimensions (Supplementary Table S1 and Supplementary Figure S1). The Kaiser–Meyer–Olkin (KMO) value was 0.793 (*p* < 0.001), indicating adequate sampling suitability. Detailed CFA results are provided in the Supplementary material.

The full questionnaire is presented in Supplementary material. The final questionnaire encompassed three primary sections, i.e., demographic information, and the knowledge and attitude dimensions; scoring criteria and details are provided in Supplementary material, with a score >70% of the maximum in each section denoting adequate knowledge and a positive attitude, based on commonly used cut-off criteria in KAP studies (12).

### Questionnaire distribution and quality control

This study used a convenience sampling method. Research assistants distributed paper questionnaires in the hospital’s outpatient and inpatient departments. The paper-based questionnaire was administered via face-to-face interviews by the investigators. All participants’ questions or concerns were addressed by research assistants. While efforts were made to minimize interviewer influence, such as instructing assistants to provide only procedural guidance, the possibility of subtle response bias due to the presence of research personnel during face-to-face interviews cannot be entirely excluded. This should be considered a potential limitation. After questionnaire completion, the investigators performed a preliminary review to assess completeness. Then, a designated investigator performed data entry and checked the response quality upon the collection of all questionnaires. Questionnaires with abnormal data (e.g., implausible age >150 years) or internal logical inconsistencies (e.g., reporting no chronic conditions but indicating comorbid diabetes or hypertension) were predefined for exclusion.

### Sample size

The minimal sample size was estimated based on 10 times the number of items in the knowledge and attitude sections, as previously proposed for survey studies (13). The knowledge section consisted of 20 items and the attitude section included 12 items, resulting in a total of 32 items. Therefore, the minimal required sample size was 320. Considering a 20% invalid questionnaire rate, the target sample size was set at 384. In the actual survey process, the final sample size was determined based on the number of valid questionnaires obtained after applying predefined data quality control criteria.

### Statistical analysis

SPSS 26.0 (IBM, Armonk, NY, USA) was used for descriptive statistics, group comparisons, and logistic regression analyses. AMOS 24.0 (IBM, Armonk, NY, USA) was used for examining the path analysis among education level, knowledge, and attitude. The distribution of continuous data was assessed by the Kolmogorov–Smirnov test. Continuous variables conforming to normal distribution were presented as mean ± standard deviation (SD) and compared by the Student’s t-test (two groups) or ANOVA (multiple groups). Those with skewed distribution were presented as median (range) and compared by the Wilcoxon-Mann–Whitney U-test (two groups) or the Kruskal-Wallis analysis of variance (multiple groups). Categorical variables were described as n (%) and compared by the chi-square test. Post-hoc analyses were performed to examine potential differences between predefined subgroups across the assessed variables. Univariable and multivariable logistic regression analyses were performed to determine the independent factors relevant to sufficient knowledge and positive attitude. Variables with *p* < 0.05 in univariable analysis were submitted to multivariable analysis. Multicollinearity in the multivariable logistic regression models was assessed using tolerance and variance inflation factor (VIF), with tolerance >0.1 and VIF < 5 indicating no serious multicollinearity. In the path analysis, standardized path coefficients, 95% confidence intervals, and *p* values were reported. Because the path model was a simple just-identified/saturated model, conventional global model-fit indices were not informative and therefore were not used as the primary basis for model evaluation. A two-sided *p* < 0.05 was considered statistically significant.

## Results

A total of 362 questionnaires were initially collected, of which 1 reported a participant age <18 years, 1 had incomplete data or logical errors, and 35 had “uncertain” as a choice for all knowledge-related questions. After the exclusions, 325 valid questionnaires were included for analysis, with a mean participant age of 31.87 ± 4.21 years. Among the participants, 212 (65.23%) lived in urban areas, 206 (63.38%) had a college/bachelor’s degree, and 159 (48.93%) had a family monthly income of 5,000–10,000 Yuan. Additionally, 258 (79.38%) participants had regular menstruation before pregnancy, 121 (37.23%) had previous miscarriage, 175 (53.85%) had first-time delivery, 206 (63.38%) had first-time cesarean section and 43 (13.23%) underwent other uterine surgeries. Concurrently, 67 (20.62%) had underlying diseases before pregnancy, and 120 (36.92%) had underlying diseases during pregnancy (Table 1).

**Table 1:** Baseline features and KA scores.

Characteristics	*N* (%)	Knowledge, means ± SD	*P*	Attitude, mean ± SD	P
Total score		11.14 ± 4.34		49.88 ± 5.79	
Age	31.87 ± 4.21				
Residence			0.006		0.037
Rural	79 (24.31)	10.04 ± 4.42		49.62 ± 6.59	
Urban	212 (65.23)	11.66 ± 4.35		50.25 ± 5.55	
Suburban	34 (10.46)	10.50 ± 3.60		48.18 ± 5.03	
Education level			<0.001		0.002
Junior high school or below	37 (11.38)	8.97 ± 4.27		46.35 ± 6.12	
High school/vocational high school	63 (19.39)	10.84 ± 3.87		49.41 ± 6.35	
Junior college/bachelor’s degree	206 (63.38)	11.36 ± 4.45		50.61 ± 5.39	
Master’s degree or above	19 (5.85)	14.05 ± 2.68		50.42 ± 5.10	
Family monthly income (CNY)			0.640		0.472
<5,000	52 (16.00)	10.69 ± 4.08		48.94 ± 6.76	
5,000–10,000	159 (48.93)	11.36 ± 4.42		49.90 ± 5.73	
10,000–20,000	82 (25.23)	10.99 ± 4.40		50.54 ± 5.29	
>20,000	32 (9.85)	11.19 ± 4.38		49.66 ± 5.62	
Regular menstruation before pregnancy			0.791		0.809
Yes	258 (79.38)	11.14 ± 4.40		49.86 ± 5.60	
No	67 (20.62)	11.16 ± 4.16		49.96 ± 6.50	
Previous miscarriage			0.813		0.373
Yes	121 (37.23)	11.29 ± 3.96		49.57 ± 5.88	
No	204 (62.77)	11.06 ± 4.56		50.07 ± 5.74	
First-time delivery			0.968		0.236
Yes	175 (53.85)	11.13 ± 4.49		50.24 ± 5.89	
No	150 (46.15)	11.16 ± 4.18		49.47 ± 5.66	
First-time cesarean section			0.563		0.246
Yes	206 (63.38)	11.20 ± 4.48		50.16 ± 5.87	
No	119 (36.62)	11.04 ± 4.11		49.41 ± 5.64	
Other uterine surgeries			0.566		0.721
Yes	43 (13.23)	11.47 ± 4.29		49.56 ± 6.27	
No	282 (86.77)	11.10 ± 4.36		49.93 ± 5.72	
Underlying diseases before pregnancy			0.141		0.279
Yes	67 (20.62)	10.51 ± 4.17		50.66 ± 5.90	
No	258 (79.38)	11.31 ± 4.38		49.68 ± 5.75	
Underlying diseases during pregnancy			0.540		0.721
Yes	120 (36.92)	11.05 ± 4.05		50.01 ± 5.68	
No	205 (63.08)	11.20 ± 4.52		49.81 ± 5.86	

Mean knowledge and attitude scores were 11.14 ± 4.34 (0–20) and 49.88 ± 5.79 (12–60), respectively. According to the predefined cut-off (>70% of the maximum score), 85 participants (26.15%) were classified as having good knowledge, and 299 participants (92.00%) were classified as having positive attitudes toward CSP. Urban residents (*p* = 0.006) and highly educated individuals (*p* < 0.001) had elevated knowledge scores. In addition, the urban group (*p* = 0.037) and women with higher education (p < 0.001 and *p* = 0.002) were more likely to exhibit higher attitude scores (Table 1).

In this study, two knowledge items had the highest correctness rates, i.e., “*Cesarean scar pregnancy can lead to uterine rupture and bleeding, posing a serious threat to maternal and fetal health*.” (K5) and “*History of uterine surgery increases the risk of cesarean scar pregnancy*” (K3-2), with 80.92 and 80.00%, respectively. Items with the lowest correctness rates included “*Inadequate nutritional intake during pregnancy increases the risk of cesarean scar pregnancy*” (K3-3) and “*Poor emotional well-being during pregnancy increases the risk of cesarean scar pregnancy*” (K3-4) with 28.92 and 30.46%, respectively (Table 2). These findings suggest that participants had relatively better awareness of the general risks and consequences of CSP, while important gaps remained in understanding specific risk factors, treatment options, and management strategies. In particular, misconceptions were observed regarding modifiable and non-modifiable risk factors, as well as the necessity, timing, and type of clinical intervention. Furthermore, moderate correctness rates for items related to treatment approaches and clinical management (e.g., K7–K10) indicate that participants had limited understanding of appropriate therapeutic strategies for CSP.

**Table 2:** Knowledge dimension.

Items, n (%)	Correctness rate, *N* (%)
1. Cesarean scar pregnancy refers to the embryo implanting at the site of a previous uterine surgical scar during pregnancy.	191 (58.77)
2. Pregnancy in the scarred area after cesarean section is the most common cause of cesarean scar pregnancy.	200 (61.54)
3. Which of the following conditions increases the risk of cesarean scar pregnancy:	
3.1 Multiple miscarriages or previous childbirth experiences	241 (74.15)
3.2 History of uterine surgery	260 (80.00)
3.3 Inadequate nutritional intake during pregnancy	94 (28.92)
3.4 Poor emotional well-being during pregnancy	99 (30.46)
4. Early cesarean scar pregnancy exhibits signs and symptoms similar to a normal pregnancy.	178 (54.77)
5. Cesarean scar pregnancy can lead to uterine rupture and bleeding, posing a serious threat to maternal and fetal health.	263 (80.92)
6. Treatment options for cesarean scar pregnancy include surgery and medication.	204 (62.77)
7. Early cesarean scar pregnancy can be addressed through medication.	125 (38.46)
8. Cesarean scar pregnancy generally may naturally resolve with conservative treatment and may not require intervention.	139 (42.77)
9. Surgical treatment for cesarean scar pregnancy may involve the removal of the uterus.	99 (30.46)
10. Once diagnosed with cesarean scar pregnancy during pregnancy, termination, and removal of the gestational tissue should be done promptly.	132 (40.62)
11. Diagnosis of cesarean scar pregnancy usually requires confirmation through ultrasound examination.	228 (70.15)
12. Cesarean scar pregnancy may require long-term monitoring and observation for confirmation.	179 (55.08)
13. Pregnant women diagnosed with cesarean scar pregnancy typically require surgical intervention.	163 (50.15)
14. The incidence of cesarean scar pregnancy does not increase with an increase in the number of uterine surgeries.	148 (45.54)
15. The occurrence of cesarean scar pregnancy is related to the recovery status of the uterus, and early pregnancy after surgery is not advisable.	255 (78.46)
16. For post-cesarean section women without fertility requirements, long-term and effective contraceptive methods should be used to prevent cesarean scar pregnancy.	221 (68.00)
17. Patients with cesarean scar pregnancy, accompanied by penetrating placental implantation and uterine rupture, may exhibit symptoms such as abdominal pain, shock, and loss of fetal heartbeats.	203 (62.46)

For most attitude items, the participants’ responses were positive. Specifically, 77.54% of participants strongly agreed that it is necessary to know in advance the risks and precautions against CSP after cesarean section (A1), and 65.23% were very willing to learn more about it to prepare for the next healthy pregnancy (A10). Moreover, 36.92% of women agreed that timely treatment of CSP in the hospital is sufficient rather than terminating the pregnancy (A7). It is interesting to note that when it comes to CSP is a low probability event (A12), 20.31% of participants agreed, 28.92% disagreed and 20.62% were neutral (Table 3).

**Table 3:** Attitude dimension.

Items, n (%)	Strongly agree	Agree	Neutral	Disagree	Strongly disagree
1. I believe it is necessary to proactively understand the risks and preventive measures of cesarean scar pregnancy after a cesarean section.	252 (77.54)	64 (19.69)	6 (1.85)	2 (0.62)	1 (0.31)
2. I consider cesarean scar pregnancy to be a very serious pregnancy condition.	161 (49.54)	97 (29.85)	42 (12.92)	25 (7.69)	/
3. I worry about the possibility of cesarean scar pregnancy when considering another pregnancy.	165 (50.77)	115 (35.38)	34 (10.46)	9 (2.77)	2 (0.62)
4. I believe that a subsequent pregnancy involving a scarred uterus poses a significant risk to both the mother and the fetus, potentially threatening their lives.	174 (53.54)	115 (35.38)	23 (7.08)	11 (3.38)	2 (0.62)
5. I am willing to adopt contraceptive measures to avoid another pregnancy.	184 (56.62)	106 (32.62)	28 (8.62)	7 (2.15)	
6. If planning another pregnancy, I would proactively develop my pregnancy plan under the guidance of a doctor.	208 (64)	102 (31.38)	12 (3.69)	2 (0.62)	1 (0.31)
7. I believe that timely treatment at the hospital is sufficient for cesarean scar pregnancy and may not necessarily require pregnancy termination.	116 (35.69)	120 (36.92)	43 (13.23)	33 (10.15)	13 (4)
8. I think scar uterus pregnancy requires an individualized approach.	157 (48.31)	130 (40)	32 (9.85)	5 (1.54)	1 (0.31)
9. The final decision after the occurrence of cesarean scar pregnancy should be made by the pregnant woman herself.	128 (39.38)	95 (29.23)	42 (12.92)	49 (15.08)	11 (3.38)
10. I am willing to learn more about relevant knowledge to prepare for a healthy pregnancy in the future.	212 (65.23)	101 (31.08)	12 (3.69)	/	/
11. Whether to conceive again depends on my pregnancy needs, and cesarean scar pregnancy does not affect my attitude toward another pregnancy.	92 (28.31)	82 (25.23)	59 (18.15)	72 (22.15)	20 (6.15)
12. I believe cesarean scar pregnancy is a low-probability event and is unlikely to occur.	57 (17.54)	66 (20.31)	67 (20.62)	94 (28.92)	41 (12.62)

Univariate and multivariate logistic regression analyses showed that having a junior college/bachelor’s degree (OR = 4.742, 95%CI: 1.399–16.069; *p* = 0.012) and having a Master’s degree or above (OR = 9.981, 95%CI: 2.251–44.255; *p* = 0.002) were independently associated with sufficient knowledge (Table 4). Meanwhile, having a high school/vocational high school education (OR = 3.534, 95%CI: 1.224–10.198; *p* = 0.020) and junior college/bachelor’s degree (OR = 12.352, 95%CI: 4.373–34.887; *p* < 0.001) were independently associated with a positive attitude (Table 5). Multicollinearity diagnostics showed no serious multicollinearity in the multivariable logistic regression models, with all tolerance values >0.1 and all VIF values <5 (Supplementary Table S2).

**Table 4:** Univariate and multivariate logistic regression analyses of knowledge.

Characteristics	Univariate logistic regression		Multivariate logistic regression	
	OR (95%CI)	*P*	OR (95%CI)	*P*
**Age**	0.978 (0.921–1.038)	0.458		
Residence				
Rural	ref			
Urban	1.542 (0.836–2.844)	0.166		
Suburban	0.629 (0.211–1.871)	0.404		
Education				
Junior high school or below	ref		ref	
High school/vocational high school	2.138 (0.549–8.333)	0.273	2.191 (0.561–8.568)	0.259
Junior college/bachelor’s degree	4.993 (1.478–16.863)	0.010	4.742 (1.399–16.069)	0.012
Master’s degree or above	10.200 (2.311–45.021)	0.002	9.981 (2.251–44.255)	0.002
Monthly per capita income				
<5,000	ref			
5,000–10,000	1.557 (0.718–3.374)	0.262		
10,000–20,000	1.637 (0.706–3.797)	0.251		
>20,000	1.643 (0.584–4.623)	0.346		
Regular menstruation before pregnancy				
Yes	1.164 (0.622–2.175)	0.635		
No	ref			
Previous miscarriage				
Yes	0.580 (0.339–0.993)	0.047	0.646 (0.371–1.126)	0.123
No	ref		ref	
First-time delivery				
Yes	1.231 (0.747–2.029)	0.414		
No	ref			
First-time cesarean section				
Yes	1.545 (0.906–2.635)	0.110		
No	ref			
Other uterine surgeries				
Yes	0.836 (0.393–1.780)	0.643		
No	ref			
Underlying diseases before pregnancy				
Yes	0.553 (0.280–1.092)	0.088		
No	ref			
Underlying diseases during pregnancy				
Yes	0.736 (0.435–1.244)	0.252		
No	ref			

OR, odds ratio; CI, confidence interval; ref, reference category. The reference category (ref) for categorical variables is indicated in the appropriate row of each variable.

**Table 5:** Univariate and multivariate logistic regression analyses of attitude.

Characteristics	Univariate logistic regression		Multivariate logistic regression	
	OR (95%CI)	*P*	OR (95%CI)	*P*
Knowledge score	1.098 (1.005–1.200)	0.038	1.049 (0.949–1.159)	0.348
Age	0.967 (0.880–1.062)	0.483		
Residence				
Rural	ref			
Urban	1.480 (0.602–3.640)	0.393		
Suburban	1.164 (0.289–4.686)	0.830		
Education				
Junior high school or below	ref		ref	
High school/vocational high school	3.840 (1.351–10.914)	0.012	3.534 (1.224–10.198)	0.020
Junior college/bachelor’s degree	13.646 (4.917–37.869)	<0.001	12.352 (4.373–34.887)	<0.001
Master’s degree or above	/	0.998	/	0.998
Monthly per capita income				
<5,000	ref			
5,000–10,000	1.906 (0.708–5.129)	0.202		
10,000–20,000	3.033 (0.842–10.933)	0.090		
>20,000	1.504 (0.360–6.288)	0.576		
Regular menstruation before pregnancy				
Yes	1.808 (0.750–4.359)	0.187		
No	ref			
Previous miscarriage				
Yes	0.794 (0.352–1.789)	0.577		
No	ref			
First-time delivery				
Yes	1.182 (0.530–2.636)	0.682		
No	ref			
First-time cesarean section				
Yes	1.538 (0.687–3.445)	0.295		
No	ref			
Other uterine surgeries				
Yes	0.611 (0.218–1.718)	0.351		
No	ref			
Underlying diseases before pregnancy				
Yes	1.468 (0.488–4.415)	0.494		
No	ref			
Underlying diseases during pregnancy				
Yes	0.931 (0.408–2.123)	0.865		
No	ref			

OR, odds ratio; CI, confidence interval; ref, reference category. The reference category (ref) for categorical variables is indicated in the appropriate row of each variable.

Post-hoc pairwise comparisons with Bonferroni correction were conducted to further explore differences in knowledge and attitude scores across residence and education-level subgroups. For residence, knowledge scores differed significantly between rural and urban participants (Bonferroni-adjusted *p* = 0.011), while attitude scores differed significantly between urban and suburban participants (Bonferroni-adjusted *p* = 0.041). For education level, several differences in knowledge scores remained significant after correction, and attitude scores differed significantly between participants with junior high school education or below and those with junior college/bachelor’s degree (Bonferroni-adjusted *p* = 0.001) (Supplementary Table S3).

**Figure 1:**
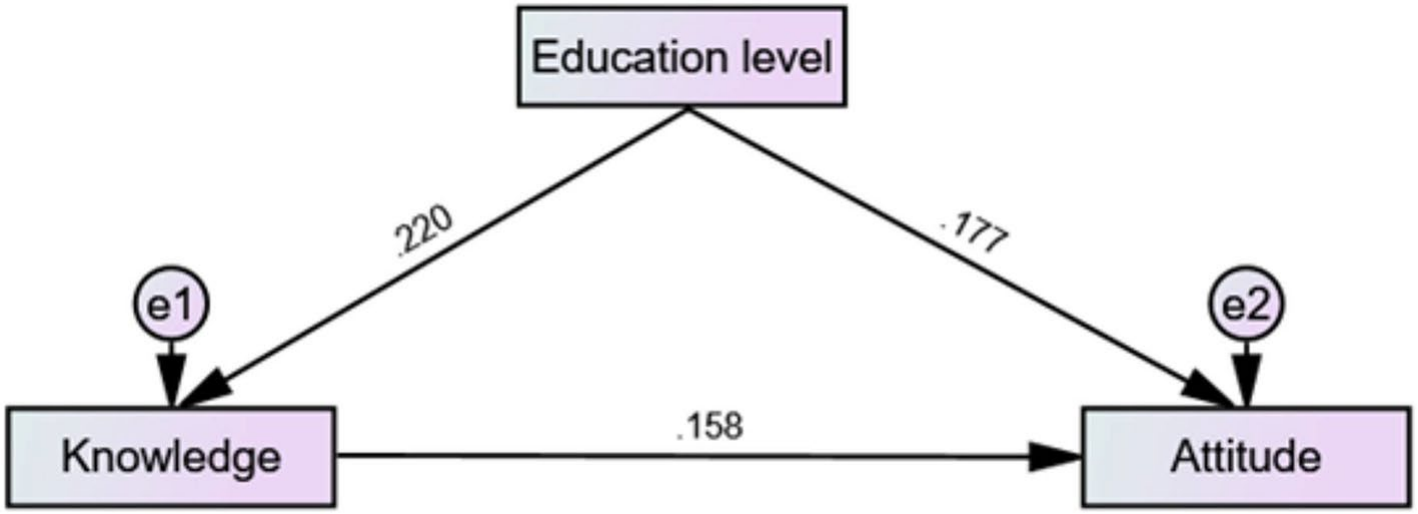
Path analysis model for the effects of education level on knowledge and attitude. Solid arrows represent significant paths, and standardized path coefficients (*β*) are displayed along each path.

Furthermore, path analysis was conducted to explore the relationships among education level, knowledge, and attitude. The model indicated that education level had a direct positive effect on knowledge (*β* = 0.220, *p* = 0.006) and attitude (β = 0.177, *p* = 0.007). Knowledge also significantly influenced attitude (*β* = 0.158, *p* = 0.016). Notably, an indirect effect of education on attitude via knowledge (β = 0.035, p = 0.007) was identified, supporting the mediating role of knowledge (Table 6 and Figure 1).

**Table 6:** Path analysis of education level, knowledge, and attitude.

Model paths	Total effects		Direct effects		Indirect effects	
	*β* (95%CI)	*P*	*β* (95%CI)	*P*	*β* (95%CI)	*P*
Education → Knowledge	0.220 (0.135–0.302)	0.006	0.220 (0.135–0.302)	0.006		
Education → Attitude	0.211 (0.109–0.292)	0.008	0.177 (0.073–0.270)	0.007	0.035 (0.013–0.060)	0.007
Knowledge → Attitude	0.158 (0.047–0.233)	0.016	0.158 (0.047–0.233)	0.016		

## Discussion

Most post-cesarean section women had inadequate knowledge but generally positive attitudes toward CSP. Therefore, targeted educational activities are required to improve awareness and understanding of CSP, particularly among women with lower educational levels. It should also be noted that all participants in this study had previously undergone cesarean section, and their personal experience may have influenced their attitudes toward CSP. While such experience may enhance awareness and engagement with the topic, it may also introduce bias, as attitudes could be shaped by individual clinical experiences, including the indication for cesarean section and the degree of involvement in decision-making. Therefore, future studies may consider including pregnant women before delivery, such as those in the third trimester, to obtain a more objective assessment of attitudes toward CSP across a broader population.

Additionally, our findings underscored the effects of sociodemographic features, especially education level and place of residence, on knowledge and attitude toward CSP. Urban residents had starkly elevated knowledge scores versus rural and suburban counterparts, corroborating previous findings suggesting better access to healthcare resources and information in urban areas (14). Similarly, participants with higher education level, especially those with a Master’s degree or above, had remarkably greater knowledge scores versus less-educated counterparts, in agreement with previous reports linking higher education with better health literacy and awareness (15, 16). This emphasizes the importance of targeted educational programs aiming to enhance awareness and understanding of CSP, especially in lowly educated and rural or suburban populations. Similar patterns have been reported in other KAP studies related to ectopic pregnancy and reproductive health risks, where knowledge levels were generally limited while attitudes toward seeking care were relatively positive. For example, a recent study found that only about 37% of women had good knowledge of ectopic pregnancy, although attitudes toward early care-seeking remained favorable, with persistent misconceptions regarding causes and management (17). Another study also reported that more than half of participants had poor knowledge despite recognizing the seriousness of the condition (18). These findings are consistent with our results and suggest that knowledge gaps remain a common challenge across different populations.

The results of logistic regression analysis demonstrated significant associations of education level with both knowledge and attitude scores, suggesting education is an important determinant of health literacy and positive health behaviors. This result is not surprising. Higher education level might help develop critical thinking skills and provide access to information that would help understand and adopt positive health behaviors (19, 20). In addition, the path analysis provided further insight into the interrelationship between variables. It confirmed that knowledge partially mediated the relationship between education and attitude, which suggests that educational interventions may influence attitude indirectly by improving knowledge. This finding supports a conceptual framework where knowledge acts as a bridge between structural sociodemographic factors and individual psychological disposition.

Regarding knowledge, the examined women had relatively high awareness of some aspects of CSP, e.g., definition and potential risks according to current results of their response to each item. However, important gaps were found in their understanding of specific risk factors, therapeutic options, and management approaches for CSP. While participants were generally aware of the definition and potential severity of CSP, misconceptions persisted regarding less obvious risk factors (such as nutritional and psychological factors), as well as the necessity, timing, and type of clinical intervention. These findings highlight that, beyond overall knowledge levels, detailed domain-specific deficiencies remain and may represent critical targets for future educational interventions. This finding highlights the key focus areas for future medical education.

The response to the attitude section indicates that over 95% of the participants are willing to learn more about preparing for a healthy pregnancy. This finding also highlights the need for more in-depth medical education. To enhance knowledge among women who had undergone cesarean section, health professionals should develop and implement educational programs considering the specific needs and education level of targeted individuals (21). Such programs should deliver clear and concise information about CSP, including its definition, risk factors, symptoms, and management options, in various accessible formats, e.g., pamphlets, videos and interactive workshops (22, 23). In addition, digital health interventions, such as mobile applications or social media-based education programs, may provide scalable and cost-effective platforms for delivering tailored information, particularly for younger women. Community-based workshops and peer-support programs may be especially beneficial for women with lower education levels, helping to improve understanding through interactive and culturally appropriate approaches. Importantly, efforts should be made to dispel misconceptions and uncertainties regarding CSP, especially in terms of therapies and long-term monitoring. Furthermore, CSP education may be integrated into routine prenatal care and postpartum follow-up to ensure consistent and comprehensive knowledge dissemination throughout the reproductive health continuum (24).

While participants generally had positive attitudes toward CSP prevention and management in the present work, as reflected by the applied classification criteria, misconceptions and uncertainties persisted, especially concerning the probability of CSP occurrence and its severity. Notably, many participants were neutral or disagreed about the severity and odds of CSP occurrence, indicating the risk associated with scar uterus pregnancy may be underestimated. Furthermore, the variable attitudes toward decision-making autonomy and the perceived necessity of termination in case of CSP suggests personalized counseling and shared decision-making strategies are needed in reproductive healthcare facilities. Health professionals should stress on proactive healthcare and shared decision-making in the management of patients with CSP (25, 26). Patient counseling is required to tackle individual concerns and determine preferences about contraceptive and pregnancy management approaches (27, 28). Additionally, open communication/collaboration between health professionals and patients may provide a supportive and empowering environment resulting in informed decision-making. Furthermore, community-based interventions, e.g., support groups and peer counseling programs, is crucial for providing needed emotional support and guiding women navigate the complex process of CSP (29, 30).

Although efforts were made to achieve the target sample size, the participants were primarily drawn from a limited group, which restricts the generalizability of the findings. The use of self-reported data also introduces potential limitations, such as recall bias and social desirability bias, which may result in overestimation of knowledge or more favorable attitudes, further compounded by the absence of objective verification. Future studies may consider using anonymous or online survey methods to reduce social desirability bias and encourage more honest responses from participants. Meanwhile, although face-to-face interviews improved data completeness, the presence of research assistants during questionnaire administration might have unintentionally influenced participant responses. This potential source of bias should also be acknowledged. Additionally, as a cross-sectional study, it is not possible to establish causal relationships. The study’s focus on a specific geographic region further limits the generalizability of the data. Given that healthcare accessibility, educational background, and cultural beliefs may vary across regions in China and internationally, caution should be exercised when extrapolating these findings beyond Fujian Province. Future multi-center studies across diverse settings are warranted to validate and extend these findings. Also, this study only included post-cesarean section women, which may limit the applicability of the findings to other populations. In addition, some potentially relevant factors were not measured in this study, such as participants’ primary sources of health information, prior exposure to CSP-related education, and access to healthcare services, which may also influence knowledge and attitudes. Future research should include broader populations to enhance preventive education on CSP. Notably, although the target sample size of 384 was not reached, the final analysis included 325 valid questionnaires, which exceeded the minimum required sample size of 320 based on the study design. This difference reflects the application of strict data quality control procedures in a real-world survey setting. In addition, some subgroups, such as participants with postgraduate education, had relatively small sample sizes, which may have resulted in unstable estimates and wide confidence intervals. Therefore, the corresponding findings should be interpreted with caution.

## Conclusion

Overall, post-cesarean section women had limited knowledge and positive attitude toward CSP. Therefore, targeted educational activities are warranted to enhance awareness and understanding of CSP in this population.

## Data Availability

The original contributions presented in the study are included in the article/Supplementary material, further inquiries can be directed to the corresponding author.
