# Supplementary material for: Knowledge and attitudes toward cesarean scar pregnancy among post-cesarean section women
Source: Front Public Health. 2026 Jun 15;14:1758292. doi: 10.3389/fpubh.2026.1758292 (PMC13310905; doi:10.3389/fpubh.2026.1758292)
Supplement: Supplementary file 1 [file Data_Sheet_1.docx]

**Table S1. CFA results**

| Indicators | Reference | Actual |
| --- | --- | --- |
| CMIN/DF | 1-3: Excellent, 3-5: Good | 1.630 |
| RMSEA | <0.08: Good | 0.044 |
| IFI | >0.9: Good | 0.917 |
| TLI | >0.9: Good | 0.901 |
| CFI | >0.9: Good | 0.915 |

**Table S2. Results of the multicollinearity test**

| **Multivariate logistic regression analyses of knowledge** | **Tolerance** | **VIF** |
| --- | --- | --- |
| Education: High school/vocational high school | 0.459 | 2.180 |
| Education: Junior college/bachelor’s degree | 0.412 | 2.426 |
| Education: Master’s degree or above | 0.701 | 1.427 |
| Previous miscarriage: Yes | 0.972 | 1.028 |
| **Multivariate logistic regression analyses of attitude** | **Tolerance** | **VIF** |
| Knowledge score | 0.943 | 1.061 |
| Education: High school/vocational high school | 0.453 | 2.210 |
| Education: Junior college/bachelor’s degree | 0.403 | 2.479 |
| Education: Master’s degree or above | 0.664 | 1.505 |

**Table S3. Bonferroni-corrected post-hoc pairwise comparisons of knowledge and attitude scores by residence and education level**

|  |  | **Knowledge** | | | **Attitude** | | |
| --- | --- | --- | --- | --- | --- | --- | --- |
|  |  | **Statistic** | **P** | **Bonferroni P** | **Statistic** | **P** | **Bonferroni P** |
| Residence | Rural VS Urban | -35.912 | 0.004 | 0.011 | -14.422 | 0.243 | 0.729 |
|  | Rural VS Suburban | -3.793 | 0.843 | 1.000 | 28.336 | 0.141 | 0.422 |
|  | Urban VS Suburban | 32.119 | 0.063 | 0.190 | 42.758 | 0.014 | 0.041 |
| Education level | Junior high school or below VS High school/vocational high school | -37.083 | 0.056 | 0.336 | -42.809 | 0.027 | 0.165 |
|  | Junior high school or below VS Junior college/bachelor’s degree | -52.481 | 0.002 | 0.010 | -63.701 | <0.001 | 0.001 |
|  | Junior high school or below VS Master’s degree or above | -112.448 | <0.001 | <0.001 | -25.489 | 0.027 | 0.162 |
|  | High school/vocational high school VS Junior college/bachelor’s degree | -15.398 | 0.254 | 1.000 | -20.893 | 0.122 | 0.730 |
|  | High school/vocational high school VS Master’s degree or above | -75.365 | 0.002 | 0.013 | -15.680 | 0.523 | 1.000 |
|  | Junior college/bachelor’s degree VS Master’s degree or above | -59.967 | 0.008 | 0.045 | 5.212 | 0.817 | 1.000 |
