# Supplementary material for: Knowledge and attitudes toward cesarean scar pregnancy among post-cesarean section women
Source: Front Public Health. 2026 Jun 15;14:1758292. doi: 10.3389/fpubh.2026.1758292 (PMC13310905; doi:10.3389/fpubh.2026.1758292)
Supplement: Supplementary file 2 [file Data_Sheet_2.docx]

| Questionnaire Number: | | |
| --- | --- | --- |
| Dear Participant:  As researchers from the Fujian Provincial Maternity and Child Health Hospital, we sincerely invite you to participate in our research project. This study aims to understand the knowledge and attitudes of postpartum women who have undergone cesarean section towards subsequent pregnancies with scarred uterus, to provide a basis for the development of scientifically sound intervention strategies. This may potentially help more individuals in the future and improve their health outcomes. Your participation in this study is voluntary, and the research has been approved by the Ethics Review Committee. If you agree to participate, please refer to the following instructions:  1. Please complete the questionnaire. There are no right or wrong answers; you only need to provide information based on your actual circumstances. Feel free to raise any questions you may have during the answering process, and kindly submit the completed questionnaire promptly.  2. This study involves a simple questionnaire survey, which will not cause any harm to your physical or psychological well-being. However, it may involve some privacy issues, such as your gender and age. Rest assured that we will strictly maintain confidentiality and will not disclose your information. Please feel free to fill it out.  3. As a participant, you are entitled to stay informed about information related to this study and its progress. If you decide to withdraw from the study, please inform us, and your data will not be included in the research results.  Finally, we sincerely appreciate your time and support for our scientific research amid your busy schedule!  □I have read and agree to use the collected data for scientific research.  Signature of Informed Consent:  Date of Participation: year month day | | |
| **Part I: Basic Information** | | |
| **1. Age： ；** | |  |
| **2. Residence：** | | a. rural  b. urban  c. suburban |
| **3. Education：** | | a. primary school or below  b. junior high school  c. high school/ technical school  d. college / undergraduate  e. graduate and above |
| **4.** **In the past year, the average monthly per capita income of your household (including tangible income and rental income, etc.):______CNY** | | a.<2000  b.2000-5000  c.5000-10000  d.10000-20000  e.>20000 |
| **5.** **Was your menstrual cycle regular before pregnancy?** | | a. irregular  d. regular |
| **6.** **Number of previous miscarriages** | | a.0  b.1  c.2  d. more than 2 |
| **7. Number of childbirth：** | | a.1  b.2  c. more than 2 |
| **8.** **Number of cesarean sections:** | | a.1  b.2  c. more than 2 |
| **9.** **Have you undergone any other uterine surgeries before (such as uterine fibroids, adenomyosis, etc.)?** | | a. yes  b. no |
| **10.** **Did you have any of the following conditions before this pregnancy?** | | a. Hypertension  b. Diabetes  c. Obesity  d. Heart disease  e. Other underlying diseases |
| **11.** **Did you experience any of the following conditions during this pregnancy?** | | a. Hypertension  b. Diabetes  c. Premature birth  d. Placental abruption  e. Intrauterine growth restriction  f. Other illnesses |

| **Part II: Understanding of Scar Pregnancy** | | | |  |
| --- | --- | --- | --- | --- |
| **1. Scar pregnancy refers to the embryo implanting at the site of a previous uterine surgical scar during pregnancy.** | a. yes | b. no | c. uncertain | |
| **1. Cesarean scar pregnancy refers to the embryo implanting at the site of a previous uterine surgical scar during pregnancy.** |  |  |  |  |
| **2. Pregnancy in the scarred area after cesarean section is the most common cause of cesarean scar pregnancy.** |  |  |  |  |
| **3.** **Which of the following conditions increases the risk of cesarean scar pregnancy:** |  |  |  |  |
| **3.1 Multiple miscarriages or previous childbirth experiences** |  |  |  |  |
| **3.2 History of uterine surgery** |  |  |  |  |
| **3.3 Inadequate nutritional intake during pregnancy** |  |  |  |  |
| **3.4 Poor emotional well-being during pregnancy** |  |  |  |  |
| **4. Early cesarean scar pregnancy exhibits signs and symptoms similar to a normal pregnancy.** |  |  |  |  |
| **5. Cesarean scar pregnancy can lead to uterine rupture and bleeding, posing a serious threat to maternal and fetal health.** |  |  |  |  |
| **6. Treatment options for cesarean scar pregnancy include surgery and medication.** |  |  |  |  |
| **7. Early cesarean scar pregnancy can be addressed through medication.** |  |  |  |  |
| **8. Cesarean scar pregnancy generally may naturally resolve with conservative treatment and may not require intervention.** |  |  |  |  |
| **9. Surgical treatment for cesarean scar pregnancy may involve the removal of the uterus.** |  |  |  |  |
| **10. Once diagnosed with cesarean scar pregnancy during pregnancy, termination, and removal of the gestational tissue should be done promptly.** |  |  |  |  |
| **11. Diagnosis of cesarean scar pregnancy usually requires confirmation through ultrasound examination.** |  |  |  |  |
| **12. Cesarean scar pregnancy may require long-term monitoring and observation for confirmation.** |  |  |  |  |
| **13. Pregnant women diagnosed with cesarean scar pregnancy typically require surgical intervention.** |  |  |  |  |
| **14. The incidence of cesarean scar pregnancy does not increase with an increase in the number of uterine surgeries.** |  |  |  |  |
| **15. The occurrence of cesarean scar pregnancy is related to the recovery status of the uterus, and early pregnancy after surgery is not advisable.** |  |  |  |  |
| **16. For post-cesarean section women without fertility requirements, long-term and effective contraceptive methods should be used to prevent cesarean scar pregnancy.** |  |  |  |  |
| **17. Patients with cesarean scar pregnancy, accompanied by penetrating placental implantation and uterine rupture, may exhibit symptoms such as abdominal pain, shock, and loss of fetal heartbeats.** |  |  |  |  |
| **2. Pregnancy in the scarred area after cesarean section is the most common cause of scar pregnancy.** | a. yes | b. no | c. uncertain | |
| **3.** **Which of the following conditions increases the risk of scar pregnancy:** |  |  |  | |
| **3.1 Multiple miscarriages or previous childbirth experiences** | a. yes | b. no | c. uncertain | |
| **3.2 History of uterine surgery** | a. yes | b. no | c. uncertain | |
| **3.3 Inadequate nutritional intake during pregnancy** | a. yes | b. no | c. uncertain | |
| **3.4 Poor emotional well-being during pregnancy** | a. yes | b. no | c. uncertain | |
| **4. Early scar pregnancy exhibits signs and symptoms similar to a normal pregnancy.** | a. yes | b. no | c. uncertain | |
| **5. Scar pregnancy can lead to uterine rupture and bleeding, posing a serious threat to maternal and fetal health.** | a. yes | b. no | c. uncertain | |
| **6. Treatment options for scar pregnancy include surgery and medication.** | a. yes | b. no | c. uncertain | |
| **7. Early scar pregnancy can be addressed through medication.** | a. yes | b. no | c. uncertain | |
| **8. Scar pregnancy generally may naturally resolve with conservative treatment and may not require intervention.** | a. yes | b. no | c. uncertain | |
| **9. Surgical treatment for scar pregnancy may involve the removal of the uterus.** | a. yes | b. no | c. uncertain | |
| **10. Once diagnosed with scar pregnancy during pregnancy, termination, and removal of the gestational tissue should be done promptly.** | a. yes | b. no | c. uncertain | |
| **11. Diagnosis of scar pregnancy usually requires confirmation through ultrasound examination.** | a. yes | b. no | c. uncertain | |
| **12. Scar pregnancy may require long-term monitoring and observation for confirmation.** | a. yes | b. no | c. uncertain | |
| **13. Pregnant women diagnosed with scar pregnancy typically require surgical intervention.** | a. yes | b. no | c. uncertain | |
| **14. The incidence of scar pregnancy does not increase with an increase in the number of uterine surgeries.** | a. yes | b. no | c. uncertain | |
| **15. The occurrence of scar pregnancy is related to the recovery status of the uterus, and early pregnancy after surgery is not advisable.** | a. yes | b. no | c. uncertain | |
| **16. For post-cesarean section women without fertility requirements, long-term and effective contraceptive methods should be used to prevent scar pregnancy.** | a. yes | b. no | c. uncertain | |
| **17. Patients with scar pregnancy, accompanied by penetrating placental implantation and uterine rupture, may exhibit symptoms such as abdominal pain, shock, and loss of fetal heartbeats.** | a. yes | b. no | c. uncertain | |

Red font, the correct answer to the question.

| **Part III: Attitudes towards Scar Pregnancy** | | | | | |
| --- | --- | --- | --- | --- | --- |
| **1. I believe it is necessary to proactively understand the risks and preventive measures of cesarean scar pregnancy after a cesarean section.** | a. strongly agree | b. agree | c. neutral | d. disagree | e. strongly disagree |
| **2. I consider cesarean scar pregnancy to be a very serious pregnancy condition.** | a. strongly agree | b. agree | c. neutral | d. disagree | e. strongly disagree |
| **3. I worry about the possibility of cesarean scar pregnancy when considering another pregnancy.** | a. strongly agree | b. agree | c. neutral | d. disagree | e. strongly disagree |
| **4. I believe that a subsequent pregnancy involving a scarred uterus poses a significant risk to both the mother and the fetus, potentially threatening their lives.** | a. strongly agree | b. agree | c. neutral | d. disagree | e. strongly disagree |
| **5. I am willing to adopt contraceptive measures to avoid another pregnancy.** | a. strongly agree | b. agree | c. neutral | d. disagree | e. strongly disagree |
| **6. If planning another pregnancy, I would proactively develop my pregnancy plan under the guidance of a doctor.** | a. strongly agree | b. agree | c. neutral | d. disagree | e. strongly disagree |
| **7. I believe that timely treatment at the hospital is sufficient for cesarean scar pregnancy and may not necessarily require pregnancy termination.** | a. strongly agree | b. agree | c. neutral | d. disagree | e. strongly disagree |
| **8. I think scar uterus pregnancy requires an individualized approach.** | a. strongly agree | b. agree | c. neutral | d. disagree | e. strongly disagree |
| **9. The final decision after the occurrence of cesarean scar pregnancy should be made by the pregnant woman herself.** | a. strongly agree | b. agree | c. neutral | d. disagree | e. strongly disagree |
| **10. I am willing to learn more about relevant knowledge to prepare for a healthy pregnancy in the future.** | a. strongly agree | b. agree | c. neutral | d. disagree | e. strongly disagree |
| **11. Whether to conceive again depends on my pregnancy needs, and cesarean scar pregnancy does not affect my attitude towards another pregnancy.** | a. strongly agree | b. agree | c. neutral | d. disagree | e. strongly disagree |
| **12. I believe cesarean scar pregnancy is a low-probability event and is unlikely to occur.** | a. strongly agree | b. agree | c. neutral | d. disagree | e. strongly disagree |
