# Supplementary figures and images for: Knowledge and attitudes toward cesarean scar pregnancy among post-cesarean section women
Source: Front Public Health. 2026 Jun 15;14:1758292. doi: 10.3389/fpubh.2026.1758292 (PMC13310905; doi:10.3389/fpubh.2026.1758292)

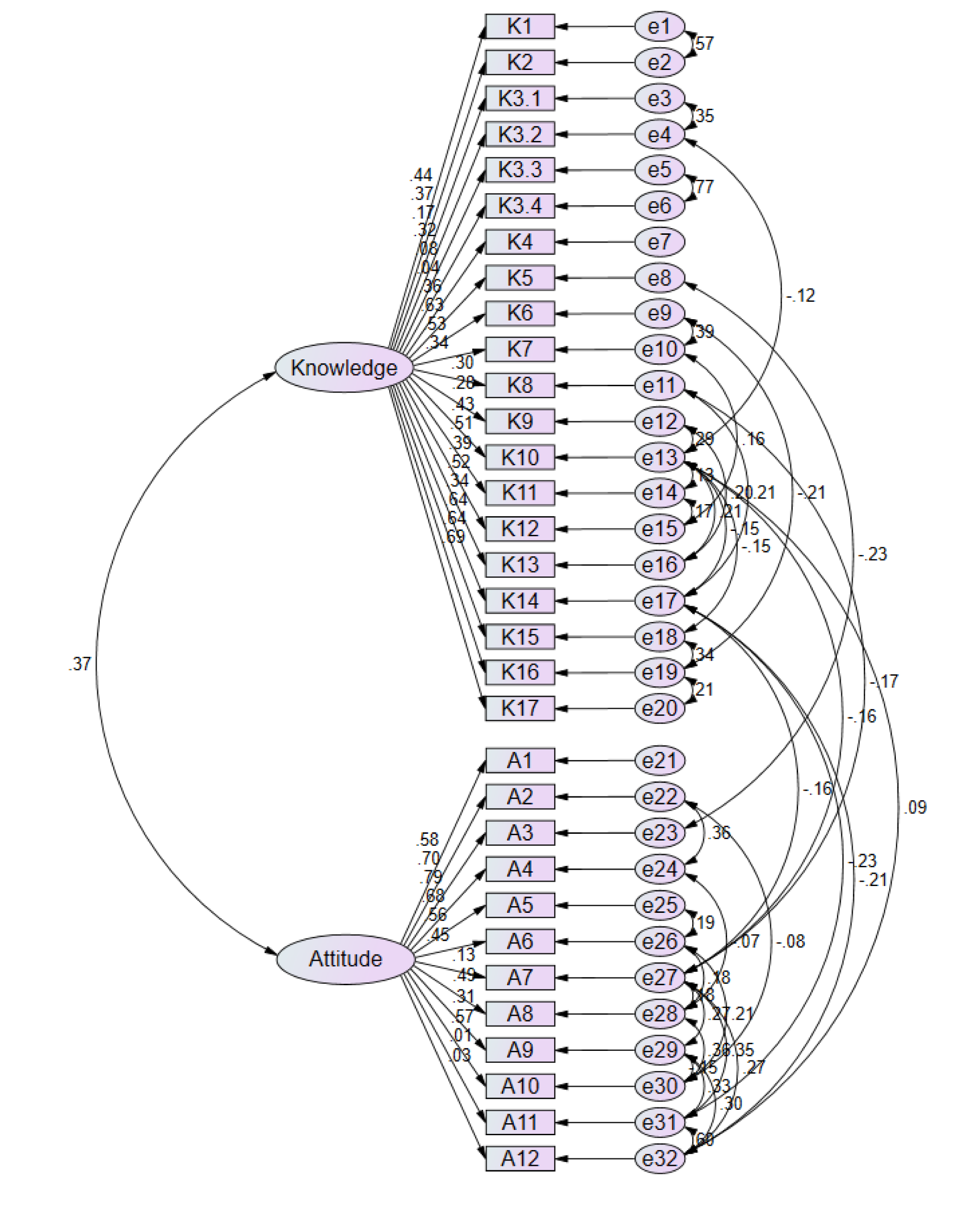

Supplement: Supplementary file 3 [file Image_1.tif]
